# Functional Studies of Novel FOXL2 Variants in Chinese Families With Blepharophimosis–Ptosis–Epicanthus Inversus Syndrome

**DOI:** 10.3389/fgene.2021.616112

**Published:** 2021-03-16

**Authors:** Fang Li, Huifang Chen, Yefei Wang, Jie Yang, Yixiong Zhou, Xin Song, Jiayan Fan

**Affiliations:** ^1^Department of Ophthalmology, Ninth People’s Hospital, Shanghai Jiao Tong University School of Medicine, Shanghai, China; ^2^Shanghai Key Laboratory of Orbital Diseases and Ocular Oncology, Shanghai, China

**Keywords:** BPES, FOXL2, forkhead domain, genotype and phenotype, POI

## Abstract

The blepharophimosis–ptosis–epicanthus inversus syndrome (BPES) is a rare autosomal dominant disease mainly caused by FOXL2 variants. This genetic disorder is usually characterized by eyelid malformation and ovarian dysfunction. However, no reliable genotype/phenotype correlations have been established considering the ovarian phenotype. Here, we detected 15 FOXL2 variants including nine novel ones from 7 families and 8 sporadic cases, which expanded the spectrum of FOXL2 variants and identified a potential clinical cause. Functional studies, with respect to the effect of FOXL2 on the StAR promoter, showed that non-sense variants that lead to protein truncation before the polyalanine tract and missense variants [c.307C > T; p.(Arg103Cys), c.311A > C; p.(His104Pro), c.320G > A; p.(Ser107Asn), and c.335T > A; p.(Phe112Tyr)] within the central portion of the FOXL2 forkhead domain significantly affect its suppressor activity. Such changes may explain the mechanism underlying a more severe phenotype, more likely to result in BPES type I. Furthermore, the missenses variants c.307C > T; p.(Arg103Cys), c.311A > C; p.(His104Pro), and c.320G > A; p.(Ser107Asn) were not able to transactivate OSR2, which is consistent with the eyelid malformation in these patients. The results from our cohort have expanded the spectrum of FOXL2 variants and have provided insights into genotype/phenotype correlations.

## Introduction

The blepharophimosis–ptosis–epicanthus inversus syndrome (BPES, MIM110100) is a rare genetic disorder that has an autosomal dominant pattern of inheritance (Beaconsfield et al., 1991). Two types of BPES have been described: type I presents with complex eyelid malformations with premature ovarian insufficiency, and type II presents only with eyelid defects (Zlotogora et al., 1983). The potential BPES locus was pinpointed in the 3q23 chromosomal region (Fukushima et al., 1991), and variants in the forkhead box L2 gene (FOXL2, OMIM# 605597) related to both types of BPES were subsequently identified (Crisponi et al., 2001).

Human FOXL2 belongs to the winged helix/forkhead transcription factor family, coding 376 amino acids, which contains a 110 amino acid DNA-binding fork head domain (forkhead domain, FHD) and a polyalanine tract of 14 residues (poly-Ala), whose expansions would cause mislocation, aggregation, and intranuclear mobility of the protein (Crisponi et al., 2001; Moumne et al., 2008; Verdin and De Baere, 2012). FHD is defined between codons 53 and 139 (pfam.xfam.org/protein/P58012), and a poly-alanine tract is between codons 221 and 234. FOXL2 is expressed in the mesenchyme of developing eyelids as well as in the granulosa cells of fetal and adult ovaries (Cocquet et al., 2002, 2003), suggesting that it might be a key regulator of eyelid formation and ovarian development and plays an important role in the vertebrate female gonad maintenance (Beysen et al., 2009; Uhlenhaut et al., 2009). FOXL2 is the first recognized ovarian differentiation marker in mammals because of its expression pattern and evolutionary conservation sequence. Furthermore, FOXL2 expression has also been identified to be important in adult pituitary development (Treier et al., 1998; Crisponi et al., 2001; Castets et al., 2020). Most patients affected by BPES only present with eyelid defects without other malformations. To date, few reports of coexisting intellectual disability are available in BPES patients with a deletion in chromosome 3q (de Ru et al., 2005) or chromosomal rearrangement (Bartholdi et al., 2008), but such a correlation was not identified in another study (Beysen et al., 2005). Thus, it is still of great interest to researchers to identify additional clinical manifestations in patients with BPES associated with FOXL2 variants.

Several genotype/phenotype correlations in BPES patients have been demonstrated after the first FOXL2 variant identification. More than 100 different FOXL2 intragenic variants are described, which are distributed along the total coding region of FOXL2. All types of variants are found including frameshift variants (44%), in-frame variants (33%), the non-sense variants (12%), and the missense variants (11%) (Verdin and De Baere, 2012). A genotype/phenotype correlation for intragenic variants has been established: variants predicted to result in truncated proteins before the poly-Ala tract might be associated with BPES type I, while variants leading to poly-Ala expansions might lead to BPES type II (De Baere et al., 2003). For variants leading to a truncated or extended protein containing an intact forkhead domain and polyalanine tract and missense variants, no clear correlations could be made. Previous studies also concluded that missense variants outside the FHD could result in a relatively mild BPES phenotype (Haghighi et al., 2012). Non-sense variants before the poly-Ala tract lead to a protein with a weak dominant negative effect as reported previously (Moumne et al., 2005). We need larger studies to demonstrate the effect of variants from different domains of FOXL2 on clinical manifestations.

In our study, we identified 15 FOXL2 variants in 7 BPES families and 8 sporadic cases, of which 9 are novel. A functional study showed that several variants were loss-of-function variants, while some others were suspected of inducing a dominant negative effect. The non-sense variants producing a protein truncation before the polyalanine tract and the missense variants [c.307C > T; p.(Arg103Cys), c.311A > C; p.(His104Pro), c.320G > A; p.(Ser107Asn), and c.335T > A; p.(Phe112Tyr)] that surrounded the p.Asn109 of FOXL2 (the central portion of the forkhead domain of FOXL2) result in lost suppression of the StAR promoter, which may affect the ovarian function. Furthermore, the missense variants [c.307C > T; p.(Arg103Cys), c.311A > C; p.(His104Pro), and c.320G > A; p.(Ser107Asn)] also inhibited luciferase activity on the OSR2 promoter, which may suggest more aggressive disruption of the protein. The patients who carried these variants [c.307C > T; p.(Arg103Cys), c.311A > C; p.(His104Pro), and c.320G > A; p.(Ser107Asn)] displayed more severe eyelid deformity. Interestingly, the patients who carried the p.(Arg103Cys) variant also displayed intellectual disability. The spectrum of FOXL2 variants has been expanded, and a potential clinical cause has been identified.

## Materials and Methods

### Subjects

A total of 136 subjects were enrolled in this study: 7 probands plus 21 other affected family members from 7 BPES families, 8 probands who were the result of sporadic cases, and 100 healthy Chinese individuals who were enrolled, including 54 unaffected relatives of the affected families. Clinical evaluations were examined by ophthalmologists, gynecologists, and maxillofacial surgeons. Patient photos in this study were taken by a hospital-based photographer in Shanghai Ninth People’s Hospital, Shanghai Jiao Tong University School of Medicine. Informed consent was obtained from all participants or their guardians for research according to the tenets of the Declaration of Helsinki and Guidance of Sample Collection of Human Genetic Diseases through the Ministry of Public Health of China.

### FOXL2 Variant Screening

Blood samples were collected from patients, relatives, and healthy volunteers. Genomic DNA was extracted from the leukocytes of peripheral venous blood of patients and healthy relatives using an Automatic Nucleic Acid Isolation System (QuickGene 800, Tokyo, Japan). PCR amplification of the genomic fragments encompassing coding regions of FOXL2 (NC_000003.12) was performed using overlapping sets of primers (Fan et al., 2011). Purified PCR products were sequenced in both directions on an ABI 3730 DNA sequencer (Applied Biosystems PerkinElmer) to confirm the variants. The variant nomenclature was used according to the Mutalyzer program^1^. Predictions of pathogenic results from missense variants were made using SIFT (Kumar et al., 2009), MutationTaster website (Schwarz et al., 2010), and Grantham score calculations (Grantham, 1974).

### Plasmid Construction

Complementary DNA (cDNA) encoding the FOXL2 open reading frame was cloned and inserted into pcDNA3.1 and EGFP-N2 vectors as previously described (Fan et al., 2011). Using the wild-type plasmids, the mutant expression vectors were obtained through PCR through different primers (Supplementary Table S1). The reporter plasmid PGL3-StAR was constructed as previously described (Fan et al., 2012). The 5,430 bp OSR2 promoter was obtained through PCR using primers with restriction enzyme sites (*Kpn*I and *Bgl*II) (Supplementary Table S1).

### Subcellular Location and Molecular Modeling

293T cells were cultured in Dulbecco’s modified Eagle’s medium (DMEM; Gibco, CA, United States) containing 10% fetal calf serum (Gibco, CA, United States) and 1% penicillin/streptomycin maintained at 37°C in a 5% CO_2_ atmosphere. As previously described, cells were seeded 24 h before transfection, and subcellular localization or aggregation was observed 48 h after transfection through confocal laser scanning microscopy using a chamber slide. To study the structural changes in the FOXL2 variants, computational analysis of the missense variant in a three-dimensional structural model was performed using Swiss-model^2^.

### Luciferase Reporter Assays

CHO cells were plated in 24-well plates and were transfected with PGL3-StAR (0.5 μg/well) and either pcDNA3.1-FOXL2-WT plasmid or one of different kinds of mutant constructs. The empty pcDNA3.1 plasmid was added as a negative control. Transfection efficiency was estimated through transfecting the PRL-TK vector that constitutively expressed Renilla luciferase, which could serve as an indicator. A dual luciferase reporter assay system (Promega) was used to conduct the luciferase assays. Supernatants of transfected cells were collected, and according to the manufacturer’s instructions on a Lumimark luminometer (Bio-Rad Laboratories, Hercules, CA, United States), luciferase activity was measured. The same method was used for transfection of PGL3-OSR2 into MLTC-1 cells and for measuring their activity. All the experiments have been performed three times and mean results are reported.

### Electrophoretic Mobility Shift Assay

The human recombinant FOXL2 protein and synthetic double-stranded oligonucleotide probes for StAR were prepared as we previously described (Fan et al., 2011; Chai et al., 2017). Protein–DNA binding reactions were performed in a 20 μl reaction system and incubated at 37°C for 30 min containing varied amounts of cell lysates and 40 ng of probe. The entire protein–oligonucleotide mixture was resolved on a 10% gel via PAGE buffered with 0.5 × TBE. ImageQuant LAS 4000 mini (GE Healthcare) was used to scan the gels.

### Statistical Analysis

The statistical analysis was performed using *t*-test. *P* < 0.05 is considered significant.

## Results

### Clinical and Genetic Features of Variants

All patients showed typical clinical features of BPES, including small palpebral fissures, ptosis, telecanthus, and epicanthus inversus. All adult female patients were examined by gynecologists, and data collected included patients’ medical history, gynecological examination, and laboratory tests. Only members of the F-2 family (III-4, III-6) [c.307C > T; p.(Arg103Cys)] were diagnosed with premature ovarian insufficiency (POI). POI in BPES is defined as amenorrhea for at least 4 months before the age of 40, combined with a decreased serum concentration of estradiol and an increased serum concentration of follicle-stimulating hormone (De Vos et al., 2010). Not all variants were evaluated with respect to POI due to the male gender and the age of the carrying patients.

**FIGURE 1:**
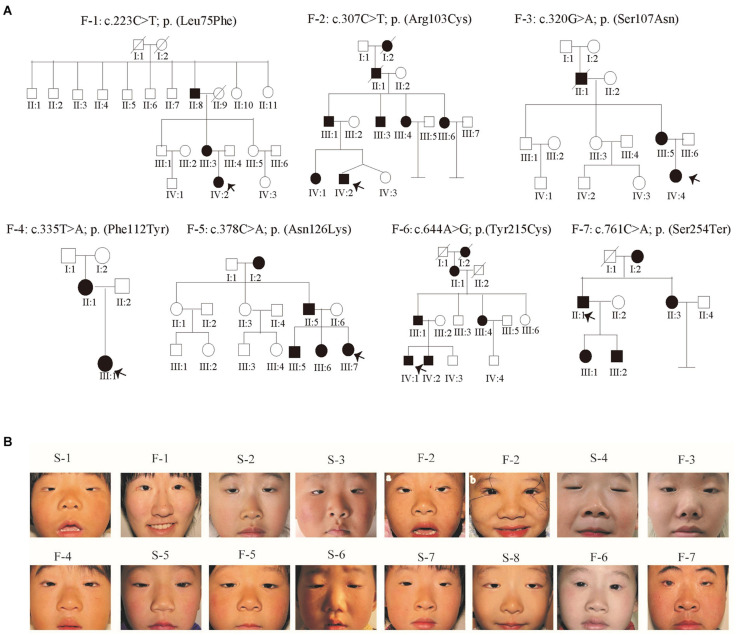
Pedigrees and facial photographs of the proband. **(A)** Pedigrees. Affected individuals are indicated by filled symbols, and the proband is marked with an upward arrow. **(B)** Photographs of the proband patients before surgery. F-1 and F-2 were photos taken after the surgery.

To better refine the genotype/phenotype correlations in our study, clinical information from all patients was collected (Table 1). In addition to the typical features of BPES, some patients also presented with flattened and broad nasal bridges. Notably, all the patients affected in family 2 also presented with intellectual disability by clinical experience. According to clinical symptoms and signs, we evaluated one BPES type I family, six BPES type II family, and eight sporadic patients (Figure 1A). BPES features were assessed through photographs of the proband taken before and/or after surgery (Figure 1B).

**TABLE 1:** Evaluation of pathogenic potential of variants in FOXL2.

Patient	Age/sex	Other anomalies		Variant	Location	Polyphen	SIFT	Grantham score	StAR binding	Activation of StAR	Repression of OSR2
		Mandibular prognathism	Flattened and broad nasal bridge								
S-1	5/F	+	+	c.173C > T; p.(Ser58Leu)	FHD	Probably damaging	Disease causing	145 (>60)	+	−	+
F-1	19/F	+	+*	c.223C > T p.(Leu75Phe)	FHD	Probably damaging	Disease causing	22	−	−	−
S-2	7/F	+	+	c.236G > T p.(Gly79Val)	FHD	Probably damaging	Disease causing	109 (>60)	+	−	+
S-3	4/M	+	+	c.293G > A p.(Trp98Ter)	FHD	N/A	Disease causing	N/A	−	+	+
F-2	4/M	+	+^#^	c.307C > T p.(Arg103Cys)	FHD	Probably damaging	Disease causing	180 (>60)	−	+	+
S-4	6/M	−	+	c.311A > C p.(His104Pro)	FHD	Probably damaging	Disease causing	77 (>60)	+	+	+
F-3	12/F	−	+	c.320G > A p.(Ser107Asn)	FHD	Probably damaging	Disease causing	46	+	+	+
F-4	9/F	−	+	c.335T > A p.(Phe112Tyr)	FHD	Probably damaging	Disease causing	22	+	+	−
S-5	7/M	−	−	c.374G > A p.(Gly125Asn)	FHD	Probably damaging	Disease causing	94 (>60)	+	−	−
F-5	9/F	−	+	c.378C > A p.(Asn126Lys)	FHD	Probably damaging	Disease causing	94 (>60)	+	−	−
S-6	6/M	−	−	c.382T > G p.(Trp128Gly)	FHD	Probably damaging	Disease causing	184 (>60)	+	−	−
S-7	7/F	−	−	c.383G > A p.(Trp128Ter)	FHD	N/A	Disease causing	N/A	−	+	−
S-8	7/M	−	−	c.415G > T p.(Gln139Ter)	FHD	N/A	Disease causing	N/A	−	+	+
F-6	7/F	−	−	c.644A > G p.(Tyr215Cys)	Between FHD and poly-Ala	Probably damaging	Disease causing	194 (>60)	+	−	−
F-7	32/M	−	−	c.761C > A p.(Ser254Ter)	Downstream of poly-Ala	N/A	Disease causing	N/A	+	−	−

*This patient had rhinoplasty at 18 age. ^#^This patient had mental retardation. F, familial; S, sporadic; “+,” present; “-,” absent; “N/A,” not applicable.

We revealed 15 FOXL2 variants in 8 sporadic cases and 7 BPES families, including 9 novel variants, 80% of which were located in the FHD of FOXL2. These variants were not found in 100 healthy control individuals. Several databases (such as NCBI, dbSNP, and UCSC) were searched to exclude variants that were, in fact, a general single-nucleotide polymorphism. Furthermore, bioinformatics software Poly-Phen, SIFT, and Grantham score calculations suggested that these variants may affect protein function (Table 1; Grantham, 1974).

### Variant Effects on Subcellular Localization

**FIGURE 2:**
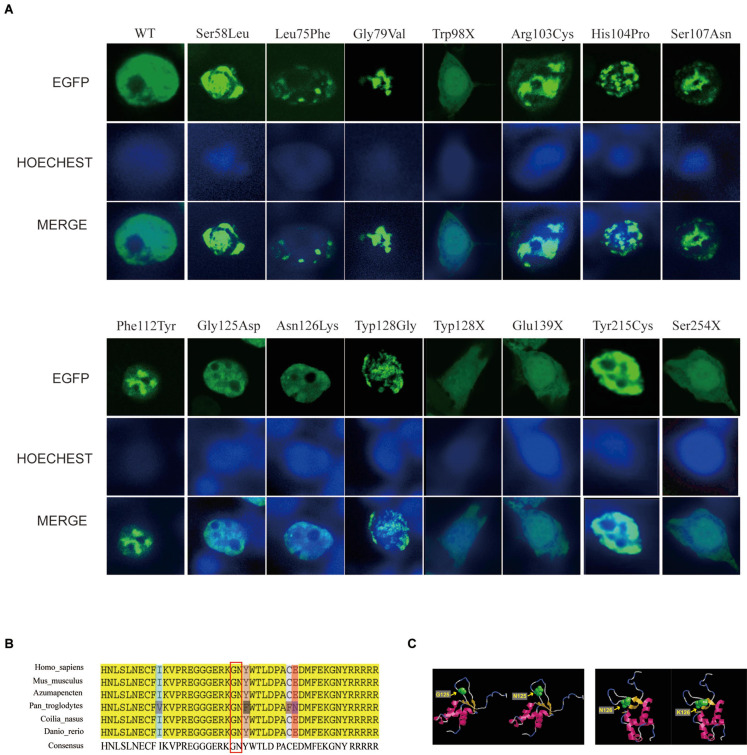
Effects of FOXL2 variants on subcellular localization. **(A)** Subcellular localization of wild-type and mutant FOXL2 protein. The first panel shows the subcellular localization of FOXL2 as a fusion protein with EGFP, and the middle panel shows the nuclear staining with HOECHST. The third panel is a merged image of the previous two images. In contrast to the wild-type FOXL2 in an exclusively nucleus localization in a diffuse manner, missense mutant protein displayed nuclear aggregation [p.(Ser58Leu), p.(Leu75Phe), p.(Gly79Val), p.(Arg103Cys), p.(His104Pro), p.(Ser107Asn), p.(Phe112Tyr), p.(Gly125Asn), and p.(Trp128Gly)], and the non-sense mutant protein showed cytoplasmic retention [p.(Trp98Ter), p.(Trp128Ter), p.(Gln139Ter), and p.(Asn254Ter)]. **(B)** Multiple alignments of six orthologs. This demonstrated high conservation of positions p.Gly125 and p.Asn126 in the FOXL2 sequence. The resource of the six orthologs was as follows: human, mouse, chlamys, chimpanzees, *Coilia nasus*, and zebrafish. **(C)** Molecular modeling of the wild-type and mutant FOXL2 [p.(Gly125Asn) and p.(Asn126Lys)] showing the position of the variants. Subtle changes were detected in the local spatial structure of the mutant FOXL2 carrying the amino acid substitution when compared with the structure of the wild-type FOXL2.

To investigate the effect of these FOXL2 variants on their function as a transcription factor, we first conducted subcellular localization studies in 293T cells. Different kinds of constructs containing EGFP-FOXL2-wild-type fusion protein (WT) or EGFP-FOXL2-mutant fusion protein were transfected into 293T cells. As shown in Figure 2, after 48 h of transfection, the wild-type FOXL2 protein and the mutants showed different localization patterns. Cells transfected with missense mutants displayed nuclear aggregation [c.173C > T; p.(Ser58Leu), c.223C > T; p.(Leu75Phe), c.236G > T; p.(Gly79Val), c.307C > T; p.(Arg103Cys), c.311A > C; p.(His104Pro), c.320G > A; p.(Ser107Asn), c.335T > A; p.(Phe112Tyr), c.374G > A; p.(Gly125Asn), and c.382T > G; p.(Trp128Gly)]. However, all the variants leading to a truncated FOXL2 protein displayed strong nuclear and cytoplasmic staining [c.383G > A; p.(Trp128Ter), c.382T > G; p.(Trp128Gly), c.415G > T; p.(Gln139Ter), and c.761C > A; p.(Ser254Ter)]. In comparison, cells transfected with WT showed an exclusively nuclear localization in a diffuse manner, to guarantee the normal interaction with its target promoters in nucleus.

These missense variants led to the mislocalization and intranuclear aggregation of the FOXL2 protein, while non-sense variants always caused protein retention in the cytoplasm. Such changes may potentially affect the interaction of FOXL2 with target promoters.

**FIGURE 3:**
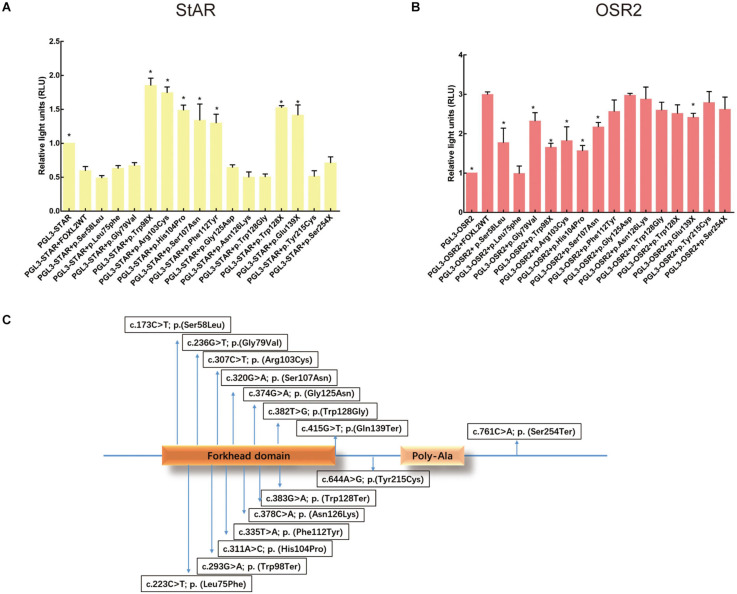
Effects of FOXL2 variant on transcriptional activity of the target genes. **(A)** StAR promoter activity as determined by reporter gene assays. As reflected by the luciferase activity, WT FOXL2 represses StAR promoter activity. However, there is no such change in luciferase activity when FOXL2 variants lead to a truncated protein before the polyalanine tract [p.(Trp128Ter), p.(Gln139Ter), and p.(Trp98Ter)] or in FOXL2 containing variants nearby the p.Asn109 [the middle position of the forkhead domain of FOXL2: p.(His104Pro), p.(Ser107Asn), and p.(Phe112Tyr)]. Statistically significant differences compared with Foxl2 WT are indicated by ^∗^*P* < 0.05. **(B)** OSR2 promoter activity determination using reporter gene assays. A luciferase reporter assay indicated that the variants [p.(Ser58Leu), p.(Leu75Phe), p.(Gly79Val), p.(Trp98Ter), p.(Arg103Cys), p.(His104Pro), and p.(Ser107Asn)] in the forkhead domain of FOXL2 inhibited luciferase activity from the OSR2 promoter. **(C)** Arrows point to the relative locations of the variants identified in the gene. The FOXL2 protein has a forkhead domain and a poly-Ala tract.

These results indicated that variants in FOXL2 may interfere with the physiological localization of the protein, indicating a change in DNA-binding and transactivation capacity. However, there was no significant difference in the subcellular localization of mutants c.374G > A; p.(Gly125Asn) and c.378C > A; p.(Asn126Lys). FOXL2 sequence alignment showed that p.Gly125 and p.Asn126 were highly conserved (Figure 3).

### Effects of FOXL2 Variant on Transcriptional Activity of the Target Genes

To test the hypothesis that certain variants alter the ability of FOXL2 to act as a transcription factor, we performed luciferase reporter analysis using two different reporter promoters that had been shown to be very sensitive that even a lower concentration of active FOXL2 could be detected (Benayoun et al., 2008; Laissue et al., 2009; Fan et al., 2011). The first one was the StAR-luc reporter (Steroidogenic Acute Regulatory), whose activity could be negatively affected by wild-type FOXL2, which is a marker of granulosa cell differentiation. The repressive activity of FOXL2 is caused by the entire alanine/proline-rich carboxyl terminus, showing a dominant negative effect. An interference of these variants with endogenous FOXL2 might evoke the expression of a reporter, but the effect was probably weak because of the overexpression of mutant FOXL2. As shown in Figure 3A, luciferase assays testing non-sense FOXL2 variants demonstrated a weak dominant negative effect on StAR, further confirming the importance of the entire alanine/proline-rich carboxyl terminus of FOXL2 in transcriptional repression of the StAR promoter. Of all missense variants, the variants p.(Arg103Cys), p.(His104Pro), p.(Ser107Asn), and p.(Phe112Tyr) all failed to inhibit the StAR activity, of which the p.(Arg103Cys) mutant was consistent with the female patients manifesting infertility in the BPES type I family (Figure 3A). On the contrary, the variants [p.(Ser58Leu), p.(Leu75Phe), p.(Gly79Val), p.(Gly125Asn); p.(Asn126Lys); p.(Trp128Gly)] inhibited StAR activity, and the patients with these variants did not present with infertility. Here, the variants c.311A > C; p.(His104Pro), c.320G > A; p.(Ser107Asn), and c.335T > A; p.(Phe112Tyr), which are nearby p.Arg103, also failed to inhibit the StAR promoter; the patients carrying these variants were either of male gender or girls in the prepubertal development stage, and we cannot therefore produce a genotype/phenotype correlation for these patients.

As OSR2 (odd-skipped related 2 transcription factor) is important in the craniofacial development process (Caburet et al., 2004), we next detected the transactivation capacity of the mutants on OSR2, which is also a target of FOXL2 (Laissue et al., 2009). As shown in Figure 3B, the missense variants [c.173C > T; p.(Ser58Leu), c.223C > T; p.(Leu75Phe), c.236G > T; p.(Gly79Val), c.307C > T; p.(Arg103Cys), c.311A > C; p.(His104Pro), and c.320G > A; p.(Ser107Asn)] close to the central portion of the FHD of FOXL2 were not able to transactivate OSR2, which is consistent with the clinical manifestations in these patients. The localization was listed in Figure 3C. However, the variants that were outside of the forkhead domain showed no significant difference from the wild-type FOXL2. In addition, the eyelid malformation of these patients was not as serious as patients carried the variant close to the carboxy terminus of FOXL2.

### Effects of the FOXL2 Variant on the Binding Ability to the StAR Promoter

**FIGURE 4:**
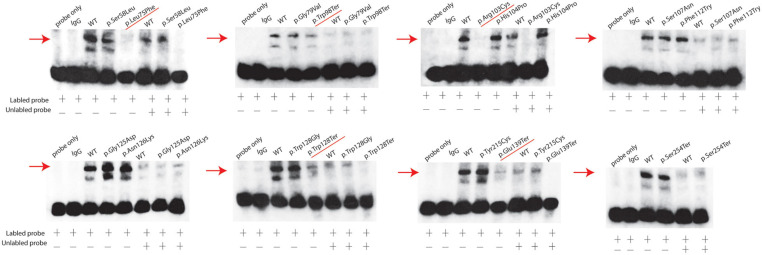
StAR promoter activity determination using EMSAs. EMSAs indicate that the wild-type FOXL2 protein was complexed with the StAR promoter. The mutant FOXL2 [p.(Leu75Phe), p.(Trp98Ter), p.(Arg103Cys), p.(Trp128Ter), and p.(Gln139Ter)] exhibited abrogated/reduced interactions. The positions of the FOXL2-StAR promoter complexes are indicated with arrows. Non-specific protein (IgG) and empty probes were used as negative controls. Probe alone is listed at the first row. A 100-fold excess of unlabeled probe was used to demonstrate specific binding between FOXL2 and the StAR promoter.

We further investigated the de-repression of the StAR promoter through electrophoretic mobility shift assay (EMSA). As shown in Figure 4, when equal amounts of mutant or wild-type FOXL2 proteins were incubated with the response element of the StAR promoter, a band shift was used to indicate the binding instead of the band corresponding to the labeled probe. To exclude off-target effects, free probe, IgG protein, and 100-fold excess of the unlabeled probe were used to further confirm the formation of the StAR-FOXL2 complex. In wild-type FOXL2, a prominent high-molecular-weight band shift indicated the binding of the protein to the StAR promoter. In contrast, when incubated with the StAR promoter, a faint band was detected in the mutant FOXL2 [c.223C > T; p.(Leu75Phe), c.293G > A; p.(Trp98Ter), c.307C > T; p.(Arg103Cys), c.383G > A; p.(Trp128Ter), and c.415G > T; p.(Gln139Ter)] group, which may be a predictor of ovarian dysfunction. Clearly, all the variants that truncated the protein before the polyalanine tract could not bind to the StAR promoter just as the wild type did, which further demonstrated that the repressor activity of FOXL2 was associated with the entire alanine/proline-rich carboxyl terminus. According to clinical examinations, ovarian insufficiency was found in one BPES type I family carrying the FOXL2 variant c.307C > T; p.(Arg103Cys).

## Discussion

BPES is a rare autosomal dominant genetic developmental disorder of the eyelids and ovary. Several variant studies have been reported since the successful cloning of the FOXL2 gene. Initially, tentative genotype/phenotype correlations were suggested, and that BPES type I might be related to truncated proteins before the polyalanine tract, and BPES type II might be related to polyalanine expansions. No correlations could be summarized for missense variants and variants leading to a truncated or extended protein containing an intact FHD and polyalanine tract. In a subsequent functional study, it was considered that missense variants were loss-of-function alleles leading to haploinsufficiency of FOXL2 (Crisponi et al., 2001). However, how the variants in the two functional domains of FOXL2 contribute to different types of clinical presentation is still unclear. Studies have reported that missense variants located in the FHD often lead to classical BPES with eyelid malformation (Beysen et al., 2008). The absence of binding to DNA could be available for protein–protein interactions, potentially leading to aggregation or cytoplasmic retention. But this could be considered as loose predictors of ovarian dysfunction. Instead, mutants completely lacking activity on reporter promoters are likely to lead to a BPES with POI (Nallathambi et al., 2008; Dipietromaria et al., 2009). But the clear-cut genotype/phenotype correlation has not been established.

Here, we reported 15 variants, including 8 novel variants in both sporadic and familial BPES patients [c.223C > T; p.(Leu75Phe), c.236G > T; p.(Gly79Val), c.320G > A; p.(Ser107Asn), c.335T > A; p.(Phe112Tyr); c.374G > A; p.(Gly125Asn); c.378C > A; p.(Asn126Lys); c.383G > A; p.(Trp128Ter), c.382T > G; p.(Trp128Gly), and c.761C > A; p.(Ser254Ter)]. Among the intragenic variants in our research, missense variants accounted for 73.3% (11/15), and 11 of them were found in the FHD domain, which is consistent with previous results (14/16) (Bunyan and Thomas, 2019). Further, the non-sense variants accounted for 26.7% (4/15), including 3 proteins truncated before the polyalanine tract.

Previous studies demonstrated that missense variants outside the FHD might result in a rather mild BPES phenotype (Haghighi et al., 2012). In comparison with these previous mild cases, our study further demonstrated that these variants in the FHD [c.307C > T; p.(Arg103Cys), c.311A > C; p.(His104Pro), and c.320G > A; p.(Ser107Asn), and c.335T > A; p.(Phe112Tyr)] that surrounded the p.Asn109 of FOXL2 (the central portion of the FHD of FOXL2) lead to a rather severe BPES phenotype in another way. A functional study showed that these variants severely impair the function of the protein, as shown by intranuclear aggregation and abnormal transcriptional activity from the StAR and OSR2 promoters. FOXL2 showed direct interaction with the promoter of StAR gene through its proline and alanine rich carboxy-terminal region to induce an obvious inhibition of its transcriptional activity, which is a marker of late differentiation of granulosa cells in pre-ovulating follicles. FOXL2 could activate the transcription of OSR2, which plays a role in the craniofacial development process. As reported by one previous study (Laissue et al., 2009), in POI patients without eyelid phenotypes, the variants could activate the reporter construct driven by the OSR2 promoter, and in BPES type I patients, the variants failed to transactivate OSR2 coherently. Interestingly, the various variants showed different behavior that some of these mutants increase and some repress StAR activity. This is suggestive of an interference of these variants with endogenous FOXL2 that might drive the expression of the reporter, which suggests the existence of a dominant negative effect. Although missense mutations affected transactivation of OSR2 more than non-sense variants, eyelid malformations were less severe [e.g., p.(Arg103Cys), p.(His104Pro), p.(Ser107Asn), compared with p.(Gln139Ter) and p.(Trp128Ter)]. This might be due to the fact that the targets of FOXL2 (with respect to eyelid malformations) may be much more than OSR2.

The p.(Arg103Cys) variant was previously reported in a sporadic 1-year-old female BPES patient. Here, apart from the ocular findings, the patients from F-2 [c.307C > T; p.(Arg103Cys)] presented with mild intellectual disability, and the female BPES patients also suffered premature ovarian insufficiency, which leads to infertility. Thus, this Chinese family was considering BPES type I. However, most BPES patients do not show an easily recognizable intellectual disability phenotype, and it is still remains to be identified whether the mental disorder in these BPES patients was also caused by the FOXL2 variant, or there were also other genetic or non-genetic familial causes. Variants c.311A > C; p.(His104Pro), c.320G > A; p.(Ser107Asn), and c.335T > A; p.(Phe112Tyr) in FOXL2 were previously reported in BPES patients. The patients who carried these variants displayed severe eye deformities, though those members had undergone successful initial medial and lateral canthoplasty (F-3-IV4, F-4-III1).

A previous study reported on the missense variant p.His104Arg in an individual with BPES (Udar et al., 2003). Here, we present the novel missense variant c.311A > C; p.(His104Pro) in one boy with BPES (S-4), whose levator function was decreased so significantly that he could not open his eyes. The eyelid phenotype was severe, showing classical BPES phenotype (i.e., blepharophimosis, ptosis, epicanthus inversus, telecanthus). Notably, the origins of the previously described families are different from ours in this study; different haplotypes and regulatory contexts were expected. The variant c.223C > T; p.(Leu75Phe) was demonstrated in an 18-year-old nulliparous woman from Poland with hormonal disorders suspected of secondary amenorrhea (Grzechocinska et al., 2019). In addition, patients carrying the variant c.644A > G; p.(Tyr215Cys) in India (Kumar et al., 2004) displayed a mild BPES phenotype, just like our patient, only showing blepharophimosis and telecanthus, but mild ptosis and epicanthus inversus.

The *in vitro* studies also contribute to the understanding of genotype/phenotype correlations through the insights into the molecular effects of FOXL2 non-sense variants. All the non-sense changes [c.383G > A; p.(Trp128Ter), c.415G > T; p.(Gln139Ter), c.293G > A; p.(Trp98Ter), and c.761C > A; p.(Ser254Ter)] were shown to result in cytoplasmic mislocalization. Furthermore, the variants leading to a protein truncation before the polyalanine tract [c.383G > A; p.(Trp128Ter), c.415G > T; p.(Gln139Ter), and c.293G > A; p.(Trp98Ter)] significantly affected FOXL2 activity on the StAR promoter. However, the patients who carried these variants were prepubertal girls or male patients, so the genotype/phenotype analysis could not be performed. Studies have found a loss-of-function allele, whose corresponding protein is unable to activate the highly sensitive 4 × FLRE-luc promoter (FOXL2 specific artificial promoter 4xFLRE-luc containing four FOXL2 response elements upstream of a minimal CMV promoter).

But all these cases emphasize the need and importance of appropriate clinical follow-up of patients carrying a protein that is truncated before the polyalanine tract to enable a timely ovarian function assessment.

In addition, in our cohort, transmission of missense variants was 11 paternal/5 maternal for the FHD and 2 paternal/3 maternal for those outside of the FHD. In Bunyan’s cohort, transmission of missense variants was 3 paternal/0 maternal for the FHD and 1 paternal/1 maternal for those outside of the FHD (Bunyan and Thomas, 2019). As for the maternal transmission difference, the sample numbers in our study is small to infer the bias. However, the results still indicated that the parental origin bias was more frequently observed in the inheritance of the familial cases, which suggests that FHD missense variants are more likely to cause ovarian insufficiency.

The spectrum of FOXL2 variants had been expanded and potential clinical causes were identified. Some variants were previously reported in Indian, Caucasian, or Iranian patients. However, the BPES phenotype of the patients reported here varied in severity, compared with previously reported cases. The variant that surrounded the middle position of the forkhead domain of FOXL2 showed more aggressive disruption of the protein than other variants. More studies are needed to identify the genotype/phenotype correlations in BPES patients.

The results from our cohort have expanded the spectrum of FOXL2 variants and have provided insights into genotype/phenotype correlations. Functional studies suggest that missense variants within the central portion of the FOXL2 forkhead domain result in a more severe phenotype and may be more likely to result in BPES type I.

## Data Availability Statement

The data presented in the study are deposited in the GenBank repository, accession numbers MW507568–MW507582.

## Ethics Statement

The studies involving human participants were reviewed and approved by the Shanghai Ninth People’s Hospital, Shanghai Jiao Tong University School of Medicine. Written informed consent to participate in this study was provided by the participants’ legal guardian/next of kin. Written informed consent was obtained from the individual(s), and minor(s)’ legal guardian/next of kin, for the publication of any potentially identifiable images or data included in this article.

## Author Contributions

JF and XS conceived the idea at the basis of the study, supervised the development of the work, and revised the manuscript. HC carried out DNA extraction and genotyping. YZ obtained the plasmids via cloning and mutagenesis and performed the functional studies. JF and FL wrote the manuscript. YW and JY helped perform the analysis with constructive discussions. All authors contributed to the article and approved the submitted version.

## Conflict of Interest

The authors declare that the research was conducted in the absence of any commercial or financial relationships that could be construed as a potential conflict of interest.

## Footnotes

1 https://mutalyzer.nl/

2 http://swissmodel.expasy.org/
