# Supplementary material for: Functional Studies of Novel FOXL2 Variants in Chinese Families With Blepharophimosis–Ptosis–Epicanthus Inversus Syndrome
Source: Front Genet. 2021 Mar 16;12:616112. doi: 10.3389/fgene.2021.616112 (PMC8007913; doi:10.3389/fgene.2021.616112)
Supplement: Supplementary file 1 [file Table_1.DOCX]

**Table S1.** **Primers**

| **Gene** | **Primer Sequence (5’-3’)** | **Purpose** |
| --- | --- | --- |
| FOXL2-F  FOXL2-R | AGCCCCCGTACTTGTACGTGGCGCTC  AAGTACGGGGGCTTCTGCGCCGGGT | construct the mutant plasmid  (S58L, c.173C>T) |
| FOXL2-F  FOXL2-R | CGGAGAAGAGGTTCACGCTGTCC  ACCTCTTCTCCGCGCTCTC | construct the mutant plasmid  (L75F, c.223C>T) |
| FOXL2-F  FOXL2-R | TCACGCTGTCCGTCATCTACCAGTAC  ACGGACAGCGTGAGCCTCTTCTC | construct the mutant plasmid  (G79V, c.236G>T) |
| FOXL2-F  FOXL2-R | GAATAAGAAGGGCTAGCAAAATAGCATCCG  TAGCCCTTCTTATTCTTCTCGTAGAACGGG | construct the mutant plasmid  (W98X, c.293G>A) |
| FOXL2-F  FOXL2-R | TGGCAAAATAGCATCTGCCACAACCTC  AGATGCTATTTTGCCAGCCCTTCTTATTC | construct the mutant plasmid  (R103C, c.307C>T) |
| FOXL2-F  FOXL2-R | AAATAGCATCCGCCCCAACCTC  GGGCGGATGCTATTTTGCCA | construct the mutant plasmid  (H104P, c.311A>C) |
| FOXL2-F  FOXL2-R | GCCACAACCTCAACCTCAACGAG  TTGAGGTTGTGGCGGATGCTATT | construct the mutant plasmid  （S107N,c.320G>A) |
| FOXL2-F  FOXL2-R | AGCCTCAACGAGTGCTACATCAAGGTG  TAGCACTCGTTGAGGCTGAGGTTGT | construct the mutant plasmid  （F112Y, c.335T>A) |
| FOXL2-F  FOXL2-R | GCGAGCGCAAGGACAACTACTGGAC  TCCTTGCGCTCGCCGCC | construct the mutant plasmid  (G125N, c.374G>A) |
| FOXL2-F  FOXL2-R | AGCGCAAGGGCAAATACTGGACGCTGG  TTTGCCCTTGCGCTCGCCGCCGCCCT | construct the mutant plasmid  (N126K, c.378C>A) |
| FOXL2-F  FOXL2-R | AAGGGCAACTACTGGACGCTGGACC  CAGTAGTTGCCCTTGCGCTCGC | construct the mutant plasmid  (W128G, c.382T>G) |
| FOXL2-F  FOXL2-R | AAGGGCAACTACTAGACGCTGGACC  TAGTAGTTGCCCTTGCGCTCGC | construct the mutant plasmid  (W128X, c.383G>A） |
| FOXL2-F  FOXL2-R | TGCGAAGACATGTTCTAGAAGGGCAAC  AGAACATGTCTTCGCAGGCCGG | construct the mutant plasmid  （Q139X,c.415G>T） |
| FOXL2-F  FOXL2-R | TCACCCATGCCCTGTGCCTCCTGCCAGATG  TAGGGCATGGGTGAGGGAGGCTG | construct the mutant plasmid  (Y215C, c.644A>G） |
| FOXL2-F  FOXL2-R | GGGCCCGGCCGCCTAGTACGGGCCGTAC  TAGGCGGCCGGGCCCGCCAGCCCCT | construct the mutant plasmid  (S254X, c.761C>A) |
| OSR2-F  OSR2-R | AGGGTACCttccagctgtcctcagac  GAAGATCTCATTCAATACAACTACCTTCACCG | construct the plasmid  PGL3-OSR2 |

The underline indicated the restriction enzyme site.
